# (1*S*,3*R*,8*R*,9*S*,11*R*)-10,10-Di­bromo-2,2-di­chloro-3,7,7,11-tetra­methyl­tetra­cyclo­[6.5.0.0^1,3^.0^9,11^]trideca­ne

**DOI:** 10.1107/S1600536813010040

**Published:** 2013-04-17

**Authors:** Abdelouahd Oukhrib, Ahmed Benharref, Mohamed Saadi, Moha Berraho, Lahcen El Ammari

**Affiliations:** a Laboratoire de Chimie Biomoléculaire, Substances Naturelles et Réactivité, Unité Associée au CNRST (URAC16)", Université Cadi Ayyad, Faculté des Sciences Semlalia, BP 2390, Bd My Abdellah, 40000 Marrakech, Morocco; b Laboratoire de Chimie du Solide Appliquée, Faculté des Sciences, Université Mohammed V-Agdal, Avenue Ibn Battouta, BP 1014, Rabat, Morocco

## Abstract

The title compound, C_17_H_24_Br_2_Cl_2_, was synthesized from β-himachalene (3,5,5,9-tetra­methyl-2,4a,5,6,7,8-hexa­hydro-1*H*-benzo­cyclo­heptene), which was isolated from the essential oil of the Atlas cedar (*Cedrus Atlantica*). The asymmetric unit contains two independent mol­ecules. Each mol­ecule is built up from fused six-, seven- and two three-membered rings. In both mol­ecules, the six-membered ring has a half-chair conformation, whereas the seven-membered ring displays a boat conformation. No specific inter­molecular inter­actions are noted in the crystal packing.

## Related literature

For similar compounds, see: Ourhriss *et al.* (2013 ▶); Oukhrib *et al.* (2013*a* ▶,*b* ▶). For the biological proprieties of β-himachalene, see: El Haib *et al.* (2011 ▶). For puckering parameters, see: Cremer & Pople (1975 ▶).
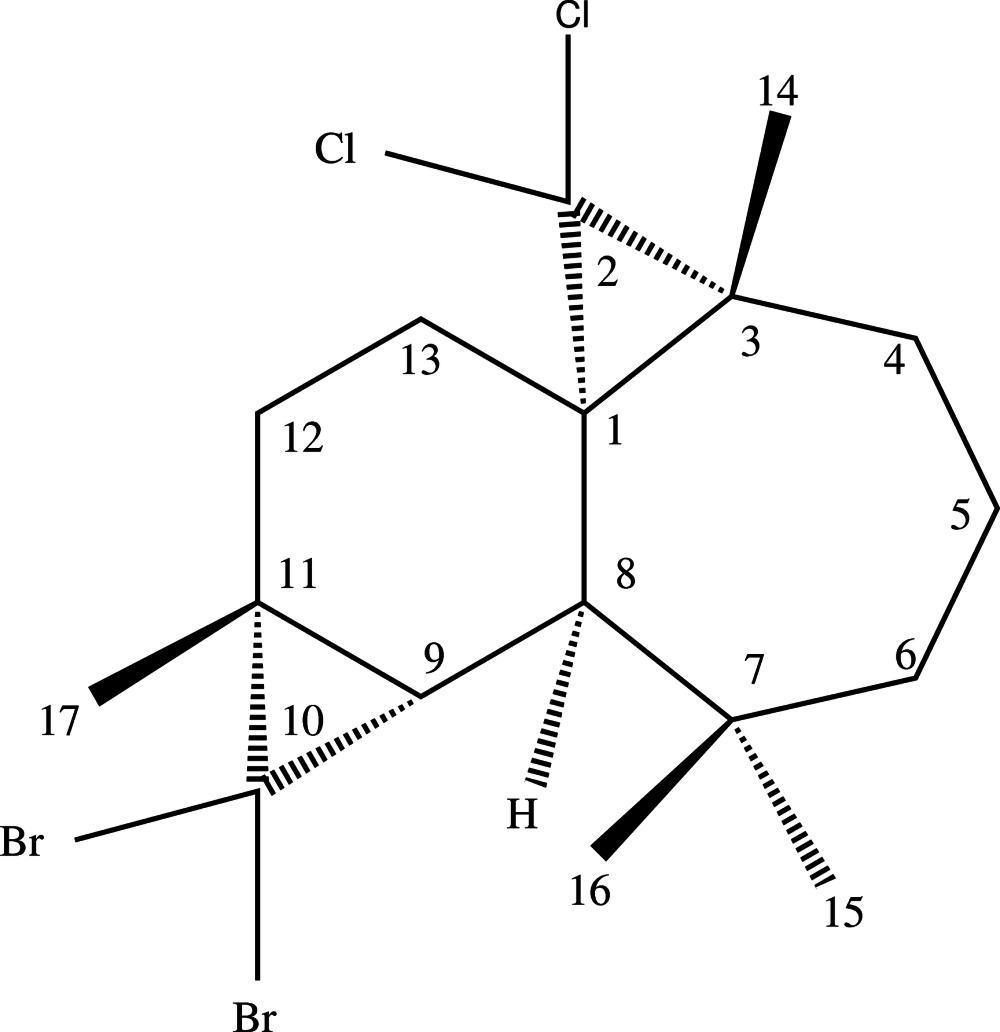


## Experimental

### 

#### Crystal data

C_17_H_24_Br_2_Cl_2_
*M* *_r_* = 459.08
Monoclinic, 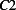

*a* = 18.377 (16) Å
*b* = 6.519 (6) Å
*c* = 30.82 (3) Å
β = 93.233 (16)°
*V* = 3687 (6) Å^3^
*Z* = 8
Mo *K*α radiation
μ = 4.68 mm^−1^
*T* = 296 K
0.43 × 0.31 × 0.27 mm

#### Data collection

Bruker X8 APEX diffractometer
Absorption correction: multi-scan (*SADABS*; Bruker, 2009 ▶) *T*_min_ = 0.739, *T*_max_ = 0.867
40489 measured reflections
9155 independent reflections
6959 reflections with *I* > 2σ(*I*)
*R*_int_ = 0.037

#### Refinement

*R*[*F* ^2^ > 2σ(*F* ^2^)] = 0.041
*wR*(*F* ^2^) = 0.103
*S* = 1.04
9155 reflections
379 parameters
1 restraint
H-atom parameters constrained
Δρ_max_ = 0.67 e Å^−3^
Δρ_min_ = −0.44 e Å^−3^
Absolute structure: Flack & Bernardinelli (2000 ▶), 3988 Friedel pairs
Flack parameter: 0.029 (8)

### 

Data collection: *APEX2* (Bruker, 2009 ▶); cell refinement: *SAINT* (Bruker, 2009 ▶); data reduction: *SAINT*; program(s) used to solve structure: *SHELXS97* (Sheldrick, 2008 ▶); program(s) used to refine structure: *SHELXL97* (Sheldrick, 2008 ▶); molecular graphics: *ORTEP-3 for Windows* (Farrugia, 2012 ▶); software used to prepare material for publication: *PLATON* (Spek, 2009 ▶) and *publCIF* (Westrip, 2010 ▶).

## Supplementary Material

Crystal structure: contains datablock(s) I, global. DOI: 10.1107/S1600536813010040/vm2192sup1.cif

Structure factors: contains datablock(s) I. DOI: 10.1107/S1600536813010040/vm2192Isup2.hkl

Supplementary material file. DOI: 10.1107/S1600536813010040/vm2192Isup3.cml

Additional supplementary materials:  crystallographic information; 3D view; checkCIF report

## Figures and Tables

**Fig. 1.**
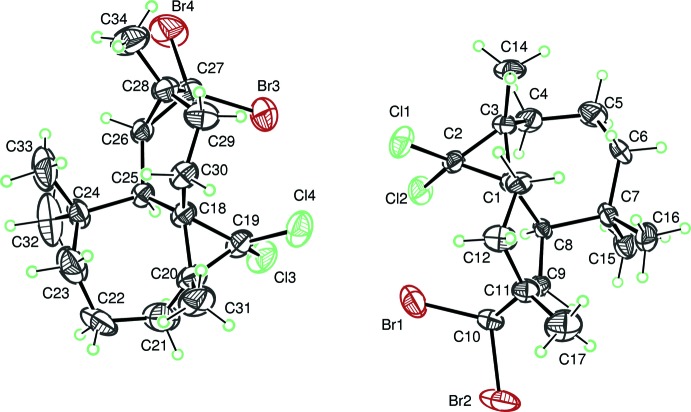
Molecular structure of the title compound with the atom-labelling scheme. Displacement ellipsoids are drawn at the 50% probability level. H atoms are represented as small spheres of arbitrary radii.

**Fig. 2.**
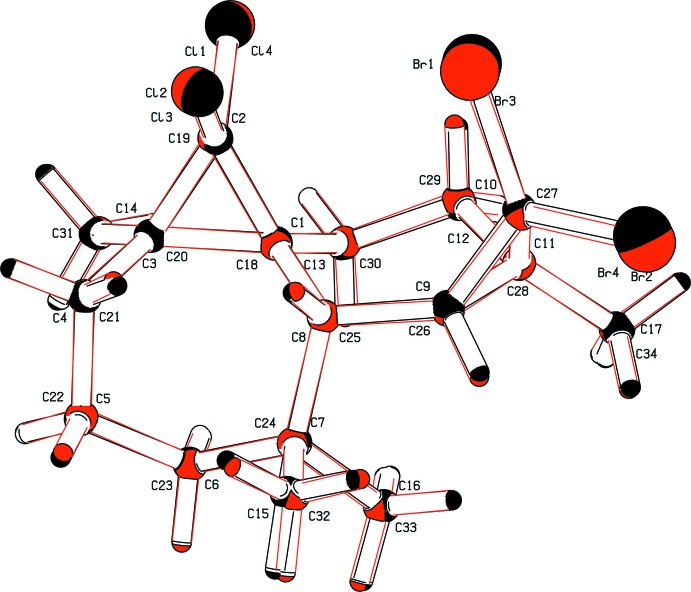
View showing the fitting of the two molecules building the asymmetric unit.

## supplementary crystallographic information

### Comment

This work is a part of our ongoing program concerning the valorization of the most abundant essential oils in Morocco, such as Cedrus atlantica. This oil is made up mainly (50%) of β-himachalene (Ourhriss *et al.*, 2013; Oukhrib *et al.*, 2013*a*,*b*). The reactivity of this sesquiterpene and its derivatives has been studied extensively by our team in order to prepare new products having biological proprieties (El Haib *et al.*, 2011). We present here the crystal structure of the title compound, (1*S*,3*R*,8*R*,9S,11*R*)-2,2-dichloro-10,10-dibromo-3,7,7,11-tetramethyltetracyclo [6.5.0.0^1,3^.0^9,11^]tridecane noted (I).

As shown in Fig. 1, each molecule of the title compound is build up by fused six- and seven-membered rings, which are fused to two three-membered rings. In the first molecule, the total puckering amplitude QT = 0.462 (4) Å and spherical polar angle θ2 = 142.4 (5)° and φ2 = 136.2 (8)°, obtained for the six-membered ring are compatible with a half chair conformation, whereas those calculated for the seven-membered ring (QT = 1.146 (5) Å, θ2 = 87.1 (3)°, φ2 = -48.8 (3)° and φ3 = -122 (4)° (Cremer & Pople, 1975)) involve a boat conformation. Nearly the same values are observed in the second molecule, and consequently the same conformation is present, as shown in the fitting drawing (Fig. 2, r.m.s. deviation 0.057 Å). Owing to the presence of Br and Cl atoms, the absolute configuration could be fully confirmed from anomalous dispersion effects, by refining the Flack parameter as C1(S),C3(*R*),C8(*R*),C9(S), and C11(*R*). The crystal packing exhibits no short intermolecular contacts.

### Experimental

A solution containing 3 g (8 mmol) of (1*S*,3*R*,8*S*)-2,2-dichloro- 3,7,7,10- tetramethyltricyclo [6.4.0.0^1,3^]dodec-9-ene and 1 ml (10 mmol) of CHBr_3_ in 40 ml of dichloromethane was added dropwise at 273 K over 30 min to 1 g of pulverized sodium hydroxide and 40 mg of *N*–benzyltriethylammonium chloride placed in a 100 ml three–necked flask. After stirring at room temperature for 2 h, the mixture was filtered on celite and concentrated in vacuum. The residue obtained was chromatographed on silicagel column impregnated with silver nitrate (10%) with a mixture of hexane - ethyl acetate (96–4) used as eluent. The two diastereoisomers (1*S*,3*R*,8*R*,9S,11*R*) -10,10-dichloro-2,2-dibromo-3,7,7,11- tetramethyltetracyclo [6.5.0.0^1,3^.0^9,11^]tridecane (*X*) and its isomer (1*S*,3*R*,8*R*, 9*R*, 11S)- 10,10- dichloro-2,2-dibromo-3,7,7,11-tetramethyltetracyclo[6.5.0.0^1,3^.0^9,11^] tridecane (Y), were obtained by this procedure in a 85/15 ratio and a combined yield of 70% (2.5 g; 5.6 mmol). The title compound (isomer *X*) was recrystallized from hexane.

### Refinement

All H atoms were fixed geometrically and treated as riding with C—H = 0.96 Å (methyl), 0.97 Å (methylene), 0.98 Å (methine) with *U*_iso_(H) = 1.2 *U*_eq_(methylene, methine) and *U*_iso_(H) = 1.5 *U*_eq_(methyl). The space group is noncentrosymmetric and the polar axis restraint is generated automatically by *SHELXL* program. The 3988 Friedel pairs were not merged.

### Crystal data

**Table:**

C_17_H_24_Br_2_Cl_2_	*F*(000) = 1840
*M**_r_* = 459.08	*D*_x_ = 1.654 Mg m^−^^3^
Monoclinic, *C*2	Mo *K*α radiation, λ = 0.71073 Å
Hall symbol: c 2y	Cell parameters from 9155 reflections
*a* = 18.377 (16) Å	θ = 2.4–28.7°
*b* = 6.519 (6) Å	µ = 4.68 mm^−^^1^
*c* = 30.82 (3) Å	*T* = 296 K
β = 93.233 (16)°	Block, colourless
*V* = 3687 (6) Å^3^	0.43 × 0.31 × 0.27 mm
*Z* = 8	

### Data collection

**Table:**

Bruker X8 APEX diffractometer	9155 independent reflections
Radiation source: fine-focus sealed tube	6959 reflections with *I* > 2σ(*I*)
Graphite monochromator	*R*_int_ = 0.037
φ and ω scans	θ_max_ = 28.7°, θ_min_ = 2.4°
Absorption correction: multi-scan (*SADABS*; Bruker, 2009)	*h* = −24→24
*T*_min_ = 0.739, *T*_max_ = 0.867	*k* = −8→8
40489 measured reflections	*l* = −41→41

### Refinement

**Table:**

Refinement on *F*^2^	Secondary atom site location: difference Fourier map
Least-squares matrix: full	Hydrogen site location: inferred from neighbouring sites
*R*[*F*^2^ > 2σ(*F*^2^)] = 0.041	H-atom parameters constrained
*wR*(*F*^2^) = 0.103	*w* = 1/[σ^2^(*F*_o_^2^) + (0.0444*P*)^2^ + 4.0898*P*] where *P* = (*F*_o_^2^ + 2*F*_c_^2^)/3
*S* = 1.04	(Δ/σ)_max_ = 0.001
9155 reflections	Δρ_max_ = 0.67 e Å^−^^3^
379 parameters	Δρ_min_ = −0.44 e Å^−^^3^
1 restraint	Absolute structure: Flack & Bernardinelli (2000), 3988 Friedel pairs
Primary atom site location: structure-invariant direct methods	Flack parameter: 0.029 (8)

### Special details

**Table:**

Geometry. All e.s.d.'s (except the e.s.d. in the dihedral angle between two l.s. planes) are estimated using the full covariance matrix. The cell e.s.d.'s are taken into account individually in the estimation of e.s.d.'s in distances, angles and torsion angles; correlations between e.s.d.'s in cell parameters are only used when they are defined by crystal symmetry. An approximate (isotropic) treatment of cell e.s.d.'s is used for estimating e.s.d.'s involving l.s. planes.
Refinement. Refinement of *F*^2^ against all reflections. The weighted *R*-factor *wR* and goodness of fit *S* are based on *F*^2^, conventional *R*-factors *R* are based on *F*, with *F* set to zero for negative *F*^2^. The threshold expression of *F*^2^ > σ(*F*^2^) is used only for calculating *R*-factors(gt) *etc*. and is not relevant to the choice of reflections for refinement. *R*-factors based on *F*^2^ are statistically about twice as large as those based on *F*, and *R*- factors based on all data will be even larger.

### Fractional atomic coordinates and isotropic or equivalent isotropic displacement parameters (Å^2^)

**Table:**

	*x*	*y*	*z*	*U*_iso_*/*U*_eq_	
Br1	0.69402 (2)	0.22377 (9)	0.666261 (16)	0.05626 (15)	
Br2	0.63800 (2)	0.29919 (8)	0.56956 (2)	0.06228 (17)	
Br3	0.81097 (3)	0.09644 (10)	0.840122 (16)	0.06037 (16)	
Br4	0.85782 (3)	0.15293 (10)	0.93871 (2)	0.06974 (19)	
Cl4	0.66339 (7)	−0.1805 (2)	0.76362 (3)	0.0535 (3)	
Cl3	0.65444 (7)	0.24769 (19)	0.78504 (4)	0.0510 (3)	
Cl2	0.85390 (6)	0.41099 (15)	0.71165 (3)	0.0384 (2)	
Cl1	0.85215 (6)	−0.01541 (18)	0.73567 (3)	0.0441 (3)	
C8	0.86557 (16)	0.2398 (5)	0.61173 (10)	0.0204 (6)	
H8	0.8641	0.3786	0.6239	0.024*	
C7	0.92400 (19)	0.2407 (6)	0.57466 (11)	0.0259 (7)	
C1	0.88476 (18)	0.0909 (5)	0.64962 (10)	0.0213 (7)	
C2	0.8812 (2)	0.1600 (6)	0.69673 (11)	0.0281 (8)	
C9	0.79025 (18)	0.1957 (5)	0.58910 (11)	0.0244 (7)	
H9	0.7874	0.2475	0.5592	0.029*	
C10	0.71591 (19)	0.1842 (6)	0.60708 (12)	0.0315 (8)	
C13	0.8639 (2)	−0.1306 (5)	0.63885 (12)	0.0279 (8)	
H13A	0.8783	−0.2171	0.6635	0.034*	
H13B	0.8906	−0.1759	0.6143	0.034*	
C12	0.7817 (2)	−0.1575 (6)	0.62784 (15)	0.0391 (10)	
H12A	0.7726	−0.2978	0.6186	0.047*	
H12B	0.7557	−0.1345	0.6539	0.047*	
C3	0.95537 (19)	0.1338 (6)	0.67808 (12)	0.0289 (8)	
C11	0.7515 (2)	−0.0120 (6)	0.59218 (12)	0.0315 (8)	
C4	0.9979 (2)	0.3277 (7)	0.66790 (12)	0.0362 (9)	
H4A	1.0303	0.3626	0.6927	0.043*	
H4B	0.9641	0.4406	0.6628	0.043*	
C16	0.9041 (2)	0.0880 (7)	0.53687 (12)	0.0400 (10)	
H16A	0.9027	−0.0491	0.5482	0.060*	
H16B	0.9402	0.0961	0.5156	0.060*	
H16C	0.8572	0.1230	0.5236	0.060*	
C15	0.9259 (2)	0.4560 (7)	0.55567 (14)	0.0439 (10)	
H15A	0.8777	0.4953	0.5453	0.066*	
H15B	0.9578	0.4580	0.5320	0.066*	
H15C	0.9436	0.5504	0.5777	0.066*	
C5	1.0427 (2)	0.2985 (8)	0.62777 (14)	0.0465 (11)	
H5A	1.0877	0.2277	0.6364	0.056*	
H5B	1.0554	0.4321	0.6166	0.056*	
C6	1.00158 (19)	0.1757 (7)	0.59140 (13)	0.0376 (10)	
H6A	0.9986	0.0349	0.6013	0.045*	
H6B	1.0318	0.1751	0.5666	0.045*	
C24	0.5723 (2)	0.0880 (7)	0.92056 (13)	0.0387 (10)	
C25	0.63553 (19)	0.0851 (6)	0.88610 (11)	0.0276 (8)	
H25	0.6389	0.2233	0.8738	0.033*	
C28	0.7494 (2)	−0.1594 (7)	0.90993 (13)	0.0382 (9)	
C14	1.0047 (2)	−0.0449 (8)	0.69381 (14)	0.0454 (11)	
H14A	1.0493	−0.0406	0.6791	0.068*	
H14B	0.9803	−0.1726	0.6876	0.068*	
H14C	1.0152	−0.0332	0.7246	0.068*	
C18	0.6206 (2)	−0.0658 (6)	0.84803 (12)	0.0319 (9)	
C20	0.5526 (2)	−0.0342 (8)	0.81676 (13)	0.0433 (10)	
C26	0.7074 (2)	0.0406 (6)	0.91218 (12)	0.0330 (9)	
H26	0.7057	0.0888	0.9422	0.040*	
C34	0.7739 (3)	−0.2710 (10)	0.95177 (16)	0.0646 (15)	
H34A	0.7455	−0.3934	0.9545	0.097*	
H34B	0.7672	−0.1830	0.9762	0.097*	
H34C	0.8245	−0.3067	0.9510	0.097*	
C30	0.6433 (2)	−0.2858 (7)	0.85972 (13)	0.0369 (9)	
H30A	0.6156	−0.3328	0.8837	0.044*	
H30B	0.6315	−0.3744	0.8350	0.044*	
C17	0.7213 (3)	−0.1179 (8)	0.55062 (16)	0.0562 (13)	
H17A	0.7494	−0.2389	0.5456	0.084*	
H17B	0.7242	−0.0259	0.5265	0.084*	
H17C	0.6714	−0.1554	0.5538	0.084*	
C21	0.5072 (3)	0.1560 (10)	0.82436 (19)	0.0658 (16)	
H21A	0.5396	0.2727	0.8287	0.079*	
H21B	0.4759	0.1826	0.7986	0.079*	
C33	0.5880 (3)	−0.0637 (10)	0.95866 (17)	0.0746 (18)	
H33A	0.5898	−0.2011	0.9475	0.112*	
H33B	0.5500	−0.0537	0.9787	0.112*	
H33C	0.6339	−0.0303	0.9733	0.112*	
C19	0.6278 (2)	−0.0030 (7)	0.80028 (13)	0.0379 (9)	
C27	0.7848 (2)	0.0368 (7)	0.89782 (13)	0.0385 (10)	
C29	0.7244 (2)	−0.3040 (7)	0.87242 (15)	0.0448 (10)	
H29A	0.7519	−0.2733	0.8473	0.054*	
H29B	0.7351	−0.4444	0.8810	0.054*	
C22	0.4606 (2)	0.1369 (10)	0.86313 (18)	0.0636 (15)	
H22A	0.4155	0.0676	0.8540	0.076*	
H22B	0.4481	0.2733	0.8729	0.076*	
C32	0.5706 (4)	0.2969 (9)	0.94119 (19)	0.079 (2)	
H32A	0.6188	0.3333	0.9523	0.118*	
H32B	0.5383	0.2949	0.9646	0.118*	
H32C	0.5538	0.3960	0.9199	0.118*	
C31	0.5073 (3)	−0.2163 (9)	0.80026 (16)	0.0604 (14)	
H31A	0.4612	−0.2156	0.8135	0.091*	
H31B	0.5328	−0.3413	0.8076	0.091*	
H31C	0.4994	−0.2071	0.7693	0.091*	
C23	0.4979 (3)	0.0178 (10)	0.90194 (17)	0.0654 (16)	
H23A	0.4649	0.0196	0.9254	0.078*	
H23B	0.5026	−0.1241	0.8930	0.078*	

### Atomic displacement parameters (Å^2^)

**Table:**

	*U*^11^	*U*^22^	*U*^33^	*U*^12^	*U*^13^	*U*^23^
Br1	0.0364 (2)	0.0805 (4)	0.0533 (3)	0.0029 (2)	0.0148 (2)	−0.0100 (3)
Br2	0.0302 (2)	0.0682 (4)	0.0871 (4)	0.0114 (2)	−0.0092 (2)	0.0232 (3)
Br3	0.0448 (3)	0.0867 (4)	0.0511 (3)	−0.0068 (3)	0.0154 (2)	0.0094 (3)
Br4	0.0436 (3)	0.0861 (5)	0.0779 (4)	−0.0139 (3)	−0.0117 (2)	−0.0230 (3)
Cl4	0.0625 (7)	0.0642 (8)	0.0342 (6)	−0.0114 (6)	0.0055 (5)	−0.0110 (5)
Cl3	0.0611 (7)	0.0489 (7)	0.0432 (6)	−0.0064 (5)	0.0051 (5)	0.0183 (5)
Cl2	0.0467 (6)	0.0344 (5)	0.0346 (5)	0.0058 (4)	0.0054 (4)	−0.0094 (4)
Cl1	0.0583 (6)	0.0463 (6)	0.0285 (5)	−0.0011 (5)	0.0087 (4)	0.0104 (4)
C8	0.0196 (15)	0.0197 (16)	0.0222 (16)	0.0038 (12)	0.0046 (12)	0.0001 (14)
C7	0.0248 (17)	0.0261 (19)	0.0275 (17)	0.0037 (14)	0.0085 (13)	0.0001 (15)
C1	0.0224 (16)	0.0195 (16)	0.0221 (16)	0.0050 (13)	0.0008 (13)	0.0007 (14)
C2	0.0334 (19)	0.0292 (19)	0.0216 (17)	0.0083 (15)	−0.0001 (14)	0.0016 (15)
C9	0.0252 (16)	0.0254 (18)	0.0228 (16)	0.0053 (14)	0.0029 (13)	0.0052 (14)
C10	0.0220 (17)	0.034 (2)	0.038 (2)	0.0016 (15)	−0.0021 (15)	0.0017 (17)
C13	0.036 (2)	0.0145 (16)	0.033 (2)	0.0073 (14)	−0.0022 (15)	0.0042 (14)
C12	0.038 (2)	0.024 (2)	0.055 (3)	−0.0049 (17)	−0.0053 (19)	0.0035 (19)
C3	0.0243 (17)	0.034 (2)	0.0276 (18)	0.0054 (15)	−0.0025 (14)	−0.0004 (16)
C11	0.0299 (19)	0.0282 (19)	0.036 (2)	−0.0007 (16)	−0.0033 (15)	−0.0016 (17)
C4	0.0292 (19)	0.042 (2)	0.037 (2)	−0.0100 (18)	−0.0069 (16)	−0.0043 (18)
C16	0.046 (2)	0.045 (2)	0.029 (2)	0.008 (2)	0.0080 (17)	−0.0047 (19)
C15	0.050 (3)	0.040 (2)	0.044 (2)	0.000 (2)	0.019 (2)	0.009 (2)
C5	0.0259 (19)	0.064 (3)	0.049 (2)	−0.008 (2)	−0.0007 (17)	0.006 (2)
C6	0.0231 (18)	0.054 (3)	0.036 (2)	0.0111 (18)	0.0109 (15)	0.0012 (19)
C24	0.030 (2)	0.048 (3)	0.040 (2)	−0.0042 (18)	0.0111 (16)	−0.005 (2)
C25	0.0291 (18)	0.0270 (19)	0.0267 (18)	−0.0070 (15)	0.0024 (14)	0.0031 (16)
C28	0.034 (2)	0.045 (3)	0.035 (2)	−0.0004 (19)	−0.0016 (17)	0.0050 (19)
C14	0.039 (2)	0.051 (3)	0.044 (2)	0.020 (2)	−0.0100 (19)	0.005 (2)
C18	0.0308 (19)	0.037 (2)	0.0280 (19)	−0.0081 (16)	−0.0006 (15)	0.0040 (17)
C20	0.038 (2)	0.055 (3)	0.036 (2)	−0.008 (2)	−0.0061 (18)	0.007 (2)
C26	0.0283 (19)	0.046 (2)	0.0245 (18)	−0.0060 (17)	0.0006 (15)	−0.0008 (17)
C34	0.066 (3)	0.069 (4)	0.057 (3)	0.001 (3)	−0.012 (3)	0.023 (3)
C30	0.044 (2)	0.032 (2)	0.035 (2)	−0.0077 (18)	−0.0008 (17)	0.0036 (18)
C17	0.051 (3)	0.055 (3)	0.061 (3)	−0.012 (2)	−0.012 (2)	−0.011 (3)
C21	0.046 (3)	0.076 (4)	0.075 (4)	0.005 (3)	0.000 (3)	0.026 (3)
C33	0.088 (4)	0.075 (4)	0.065 (3)	0.007 (3)	0.048 (3)	0.020 (3)
C19	0.042 (2)	0.042 (2)	0.030 (2)	−0.0063 (19)	−0.0020 (16)	0.0045 (18)
C27	0.033 (2)	0.050 (3)	0.032 (2)	−0.0042 (18)	−0.0054 (16)	−0.0078 (19)
C29	0.047 (2)	0.027 (2)	0.060 (3)	0.0029 (19)	−0.005 (2)	−0.002 (2)
C22	0.028 (2)	0.072 (4)	0.091 (4)	0.011 (2)	0.007 (2)	0.008 (3)
C32	0.108 (5)	0.056 (3)	0.079 (4)	−0.024 (3)	0.060 (4)	−0.020 (3)
C31	0.045 (3)	0.080 (4)	0.055 (3)	−0.023 (3)	−0.015 (2)	−0.001 (3)
C23	0.044 (3)	0.083 (4)	0.071 (4)	−0.007 (3)	0.021 (3)	−0.007 (3)

### Geometric parameters (Å, º)

**Table:**

Br1—C10	1.907 (4)	C24—C33	1.550 (7)
Br2—C10	1.940 (4)	C24—C25	1.618 (5)
Br3—C27	1.908 (4)	C25—C26	1.535 (5)
Br4—C27	1.942 (4)	C25—C18	1.544 (5)
Cl4—C19	1.768 (5)	C25—H25	0.9800
Cl3—C19	1.777 (5)	C28—C27	1.491 (6)
Cl2—C2	1.779 (4)	C28—C26	1.518 (6)
Cl1—C2	1.762 (4)	C28—C34	1.526 (6)
C8—C9	1.541 (5)	C28—C29	1.542 (6)
C8—C1	1.544 (5)	C14—H14A	0.9600
C8—C7	1.612 (4)	C14—H14B	0.9600
C8—H8	0.9800	C14—H14C	0.9600
C7—C15	1.522 (6)	C18—C30	1.531 (6)
C7—C6	1.548 (5)	C18—C19	1.541 (5)
C7—C16	1.560 (5)	C18—C20	1.549 (5)
C1—C2	1.525 (5)	C20—C19	1.514 (6)
C1—C13	1.526 (5)	C20—C31	1.520 (7)
C1—C3	1.550 (5)	C20—C21	1.520 (8)
C2—C3	1.519 (5)	C26—C27	1.513 (6)
C9—C10	1.505 (5)	C26—H26	0.9800
C9—C11	1.535 (5)	C34—H34A	0.9600
C9—H9	0.9800	C34—H34B	0.9600
C10—C11	1.519 (6)	C34—H34C	0.9600
C13—C12	1.539 (5)	C30—C29	1.523 (6)
C13—H13A	0.9700	C30—H30A	0.9700
C13—H13B	0.9700	C30—H30B	0.9700
C12—C11	1.532 (6)	C17—H17A	0.9600
C12—H12A	0.9700	C17—H17B	0.9600
C12—H12B	0.9700	C17—H17C	0.9600
C3—C4	1.528 (6)	C21—C22	1.515 (7)
C3—C14	1.537 (5)	C21—H21A	0.9700
C11—C17	1.532 (6)	C21—H21B	0.9700
C4—C5	1.536 (6)	C33—H33A	0.9600
C4—H4A	0.9700	C33—H33B	0.9600
C4—H4B	0.9700	C33—H33C	0.9600
C16—H16A	0.9600	C29—H29A	0.9700
C16—H16B	0.9600	C29—H29B	0.9700
C16—H16C	0.9600	C22—C23	1.553 (8)
C15—H15A	0.9600	C22—H22A	0.9700
C15—H15B	0.9600	C22—H22B	0.9700
C15—H15C	0.9600	C32—H32A	0.9600
C5—C6	1.541 (6)	C32—H32B	0.9600
C5—H5A	0.9700	C32—H32C	0.9600
C5—H5B	0.9700	C31—H31A	0.9600
C6—H6A	0.9700	C31—H31B	0.9600
C6—H6B	0.9700	C31—H31C	0.9600
C24—C32	1.504 (7)	C23—H23A	0.9700
C24—C23	1.524 (7)	C23—H23B	0.9700
			
C9—C8—C1	112.7 (3)	C27—C28—C26	60.4 (3)
C9—C8—C7	107.3 (3)	C27—C28—C34	120.5 (4)
C1—C8—C7	113.9 (3)	C26—C28—C34	119.8 (4)
C9—C8—H8	107.5	C27—C28—C29	116.9 (3)
C1—C8—H8	107.5	C26—C28—C29	115.3 (3)
C7—C8—H8	107.5	C34—C28—C29	113.8 (4)
C15—C7—C6	109.9 (3)	C3—C14—H14A	109.5
C15—C7—C16	108.1 (3)	C3—C14—H14B	109.5
C6—C7—C16	104.3 (3)	H14A—C14—H14B	109.5
C15—C7—C8	107.9 (3)	C3—C14—H14C	109.5
C6—C7—C8	113.6 (3)	H14A—C14—H14C	109.5
C16—C7—C8	112.9 (3)	H14B—C14—H14C	109.5
C2—C1—C13	117.6 (3)	C30—C18—C19	115.9 (3)
C2—C1—C8	121.0 (3)	C30—C18—C25	112.6 (3)
C13—C1—C8	112.7 (3)	C19—C18—C25	122.4 (3)
C2—C1—C3	59.2 (2)	C30—C18—C20	118.1 (3)
C13—C1—C3	119.0 (3)	C19—C18—C20	58.7 (3)
C8—C1—C3	117.6 (3)	C25—C18—C20	119.4 (4)
C3—C2—C1	61.2 (2)	C19—C20—C31	119.0 (4)
C3—C2—Cl1	119.8 (3)	C19—C20—C21	117.5 (4)
C1—C2—Cl1	119.2 (3)	C31—C20—C21	113.2 (4)
C3—C2—Cl2	118.2 (3)	C19—C20—C18	60.4 (3)
C1—C2—Cl2	123.1 (3)	C31—C20—C18	120.8 (4)
Cl1—C2—Cl2	108.67 (19)	C21—C20—C18	116.3 (4)
C10—C9—C11	60.0 (2)	C27—C26—C28	58.9 (3)
C10—C9—C8	130.8 (3)	C27—C26—C25	130.2 (3)
C11—C9—C8	122.9 (3)	C28—C26—C25	124.3 (3)
C10—C9—H9	111.2	C27—C26—H26	111.2
C11—C9—H9	111.2	C28—C26—H26	111.2
C8—C9—H9	111.2	C25—C26—H26	111.2
C9—C10—C11	61.0 (2)	C28—C34—H34A	109.5
C9—C10—Br1	126.2 (3)	C28—C34—H34B	109.5
C11—C10—Br1	121.4 (3)	H34A—C34—H34B	109.5
C9—C10—Br2	114.6 (3)	C28—C34—H34C	109.5
C11—C10—Br2	117.5 (3)	H34A—C34—H34C	109.5
Br1—C10—Br2	109.24 (18)	H34B—C34—H34C	109.5
C1—C13—C12	112.8 (3)	C29—C30—C18	112.5 (3)
C1—C13—H13A	109.0	C29—C30—H30A	109.1
C12—C13—H13A	109.0	C18—C30—H30A	109.1
C1—C13—H13B	109.0	C29—C30—H30B	109.1
C12—C13—H13B	109.0	C18—C30—H30B	109.1
H13A—C13—H13B	107.8	H30A—C30—H30B	107.8
C11—C12—C13	113.5 (3)	C11—C17—H17A	109.5
C11—C12—H12A	108.9	C11—C17—H17B	109.5
C13—C12—H12A	108.9	H17A—C17—H17B	109.5
C11—C12—H12B	108.9	C11—C17—H17C	109.5
C13—C12—H12B	108.9	H17A—C17—H17C	109.5
H12A—C12—H12B	107.7	H17B—C17—H17C	109.5
C2—C3—C4	117.7 (3)	C22—C21—C20	113.2 (5)
C2—C3—C14	119.4 (3)	C22—C21—H21A	108.9
C4—C3—C14	113.1 (3)	C20—C21—H21A	108.9
C2—C3—C1	59.6 (2)	C22—C21—H21B	108.9
C4—C3—C1	117.0 (3)	C20—C21—H21B	108.9
C14—C3—C1	120.1 (3)	H21A—C21—H21B	107.7
C10—C11—C17	119.4 (4)	C24—C33—H33A	109.5
C10—C11—C12	116.7 (3)	C24—C33—H33B	109.5
C17—C11—C12	114.9 (4)	H33A—C33—H33B	109.5
C10—C11—C9	59.0 (2)	C24—C33—H33C	109.5
C17—C11—C9	119.6 (4)	H33A—C33—H33C	109.5
C12—C11—C9	116.1 (3)	H33B—C33—H33C	109.5
C3—C4—C5	111.5 (4)	C20—C19—C18	60.9 (3)
C3—C4—H4A	109.3	C20—C19—Cl4	120.3 (3)
C5—C4—H4A	109.3	C18—C19—Cl4	119.5 (3)
C3—C4—H4B	109.3	C20—C19—Cl3	118.9 (3)
C5—C4—H4B	109.3	C18—C19—Cl3	122.5 (3)
H4A—C4—H4B	108.0	Cl4—C19—Cl3	108.4 (2)
C7—C16—H16A	109.5	C28—C27—C26	60.7 (3)
C7—C16—H16B	109.5	C28—C27—Br3	123.0 (3)
H16A—C16—H16B	109.5	C26—C27—Br3	124.0 (3)
C7—C16—H16C	109.5	C28—C27—Br4	117.8 (3)
H16A—C16—H16C	109.5	C26—C27—Br4	115.5 (3)
H16B—C16—H16C	109.5	Br3—C27—Br4	108.9 (2)
C7—C15—H15A	109.5	C30—C29—C28	113.1 (4)
C7—C15—H15B	109.5	C30—C29—H29A	109.0
H15A—C15—H15B	109.5	C28—C29—H29A	109.0
C7—C15—H15C	109.5	C30—C29—H29B	109.0
H15A—C15—H15C	109.5	C28—C29—H29B	109.0
H15B—C15—H15C	109.5	H29A—C29—H29B	107.8
C4—C5—C6	112.8 (3)	C21—C22—C23	113.8 (4)
C4—C5—H5A	109.0	C21—C22—H22A	108.8
C6—C5—H5A	109.0	C23—C22—H22A	108.8
C4—C5—H5B	109.0	C21—C22—H22B	108.8
C6—C5—H5B	109.0	C23—C22—H22B	108.8
H5A—C5—H5B	107.8	H22A—C22—H22B	107.7
C5—C6—C7	120.3 (4)	C24—C32—H32A	109.5
C5—C6—H6A	107.2	C24—C32—H32B	109.5
C7—C6—H6A	107.2	H32A—C32—H32B	109.5
C5—C6—H6B	107.2	C24—C32—H32C	109.5
C7—C6—H6B	107.2	H32A—C32—H32C	109.5
H6A—C6—H6B	106.9	H32B—C32—H32C	109.5
C32—C24—C23	113.1 (5)	C20—C31—H31A	109.5
C32—C24—C33	105.4 (4)	C20—C31—H31B	109.5
C23—C24—C33	102.6 (4)	H31A—C31—H31B	109.5
C32—C24—C25	108.7 (4)	C20—C31—H31C	109.5
C23—C24—C25	114.3 (3)	H31A—C31—H31C	109.5
C33—C24—C25	112.4 (4)	H31B—C31—H31C	109.5
C26—C25—C18	112.8 (3)	C24—C23—C22	119.2 (5)
C26—C25—C24	106.7 (3)	C24—C23—H23A	107.5
C18—C25—C24	113.8 (3)	C22—C23—H23A	107.5
C26—C25—H25	107.8	C24—C23—H23B	107.5
C18—C25—H25	107.8	C22—C23—H23B	107.5
C24—C25—H25	107.8	H23A—C23—H23B	107.0

## References

[bb1] Bruker (2009). *APEX2*, *SAINT* and *SADABS* Bruker AXS Inc., Madison, Wisconsin, USA.

[bb2] Cremer, D. & Pople, J. A. (1975). *J. Am. Chem. Soc.* **97**, 1354–1358.

[bb3] El Haib, A., Benharref, A., Parrès-Maynadié, S., Manoury, E., Urrutigoïty, M. & Gouygou, M. (2011). *Tetrahedron Asymmetry*, **22**, 101–108.

[bb4] Farrugia, L. J. (2012). *J. Appl. Cryst.* **45**, 849–854.

[bb5] Flack, H. D. & Bernardinelli, G. (2000). *J. Appl. Cryst.* **33**, 1143–1148.

[bb6] Oukhrib, A., Benharref, A., Saadi, M., Berraho, M. & El Ammari, L. (2013*a*). *Acta Cryst.* E**69**, o521–o522. 10.1107/S1600536813006077 PMC3629578 23634065

[bb7] Oukhrib, A., Benharref, A., Saadi, M., Berraho, M. & El Ammari, L. (2013*b*). *Acta Cryst.* E**69**, o589–o590. 10.1107/S1600536813007642 PMC3629633 23634120

[bb8] Ourhriss, N., Benharref, A., Saadi, M., El Ammari, L. & Berraho, M. (2013). *Acta Cryst.* E**69**, o275. 10.1107/S1600536813001700 PMC3569802 23424548

[bb9] Sheldrick, G. M. (2008). *Acta Cryst.* A**64**, 112–122. 10.1107/S0108767307043930 18156677

[bb10] Spek, A. L. (2009). *Acta Cryst.* D**65**, 148–155. 10.1107/S090744490804362X PMC2631630 19171970

[bb11] Westrip, S. P. (2010). *J. Appl. Cryst.* **43**, 920–925.

